# Atopic outcomes in children with Down syndrome: a pilot sibling-matched case–control study

**DOI:** 10.3389/fped.2026.1883821

**Published:** 2026-07-24

**Authors:** Nasser S. Alharbi, Ahmed Al-Eidan, Ghada Al-Ashaikh

**Affiliations:** 1 Department of Pediatrics, College of Medicine, King Saud University, Riyadh, Saudi Arabia; 2 Department of Pediatrics, King Saud University Medical City, King Saud University, Riyadh, Saudi Arabia; 3 Department of Pediatrics, Division of Pediatric Pulmonology, King Saud University Medical City, Riyadh, Saudi Arabia

**Keywords:** asthma, atopy, Down syndrome, Saudi Arabia, siblings, wheezing

## Abstract

**Introduction:**

Atopic conditions such as wheezing, asthma, allergic rhinitis, and eczema are frequently reported in children with Down syndrome. Many previous studies have used unpaired analyses that do not account for shared familial and environmental factors. Sibling-matched designs offer a robust approach for addressing these limitations but require appropriate paired statistical methods. This study aimed to assess the feasibility of a sibling-matched design for studying atopic outcomes in children with Down syndrome and to propose an appropriate methodological and statistical framework for future larger-scale studies.

**Methods:**

This pilot sibling-matched case–control study included 34 children with Down syndrome and their unaffected siblings (34 complete sibling pairs). Binary outcomes included wheezing, severe wheezing, physician-diagnosed asthma, allergic rhinitis, and eczema. Descriptive prevalence was reported, and paired comparisons were performed using exact McNemar testing based on discordant sibling pairs.

**Results:**

Wheezing and physician-diagnosed asthma were numerically more frequent among children with Down syndrome than among their unaffected siblings, but the differences did not reach statistical significance. Statistical comparisons were driven by discordant pairs, illustrating the importance of paired analytical approaches in sibling-matched studies.

**Conclusions:**

This pilot study demonstrates the feasibility of sibling-matched designs for evaluating atopic outcomes in children with Down syndrome and highlights the importance of paired statistical methods for binary outcomes. The findings primarily serve to propose a methodological framework for future adequately powered sibling-matched studies rather than to draw definitive etiological conclusions

## 1 Introduction

Down syndrome (DS), also referred to as trisomy 21, is one of the most common genetic disorders. In Saudi Arabia, a previous estimate reported the prevalence of Down syndrome as 18 per 10,000 live births, while the community-based survey by AlSalloum et al. found a Down syndrome prevalence of 6.6 per 10,000 children. The same report notes that maternal age is strongly associated with chromosomal anomalies, especially Down syndrome (1).

Individuals with trisomy 21 often encounter various health challenges, including congenital heart defects, gastrointestinal abnormalities, and endocrine disorders. Among these issues, respiratory complications resulting from anatomical differences and immune deficiencies are significant contributors to morbidity and mortality in this population (2, 3).

Research suggests that children with Down syndrome exhibit a higher prevalence of respiratory symptoms compared to their typically developing peers. A study indicated that the incidence of wheezing and respiratory symptoms was significantly elevated in children with Down syndrome, with a relative risk of current wheezing calculated at 2.8 in comparison to their siblings and 2.75 relative to controls from the general population (4). This implies that, although asthma may be diagnosed less frequently in this population, the symptoms associated with respiratory distress are more prevalent.

Asthma is a common chronic respiratory condition that can greatly affect the quality of life and health outcomes for those who have it. Children with trisomy 21 are known to experience a higher rate of respiratory problems, including asthma attributed to various anatomical and physiological factors linked to the syndrome (4, 5).

Additionally, children with trisomy 21 face unique challenges that can worsen respiratory conditions. These challenges include anatomical variations, such as hypotonia, which may result in airway obstruction, as well as an increased vulnerability to respiratory infections (5). The immune system in individuals with Down syndrome is frequently dysregulated, which may lead to heightened allergic responses and respiratory complications, this dysregulation involves abnormalities in the B- and T-cell compartments, including specific defects in B-cell memory, reduced levels of IgM, IgG2, and IgG4, impaired maturation of specific antibodies, and poor antibody responses to vaccination, together with a possible autoinflammatory component (5).

Moreover, children with Trisomy 21 do experience nasal and dermatologic conditions encountered in normally developing peers. However, allergic rhinitis may not only be explained as the cause of chronic rhinitis symptoms given the low prevalence of positive skin prick test results for aeroallergens in children with Down syndrome (6). On the other end of the atopic spectrum, the alteration of the immune system in Down syndrome makes a diagnostic challenge when evaluating atopic dermatitis. Given that, this increases their vulnerability to infections and complicate eczema management (7). Altogether, children with Down syndrome have a three-decade lower life expectancy compared with healthy children, with cardiac and respiratory illnesses being major contributors (8).

Hence, we aimed through this pilot study to screen children with Down syndrome for their respiratory and atopic profiles and compare them head-to-head with their unaffected siblings. Sibling-matched designs offer a robust approach for evaluating disease-specific risks because they inherently control for shared familial confounders, including genetic background, household environmental exposures, and socioeconomic factors. However, such designs require appropriate paired statistical methods for valid inference. Previous sibling-matched studies evaluating respiratory outcomes in Down syndrome have applied unpaired tests such as chi-square, which treat observations as independent and fail to account for within-family correlation. The present study addresses this methodological gap by applying exact McNemar testing, an appropriate paired analytical method for small sibling-matched datasets, and proposes a paired analytical framework for future larger-scale sibling-matched studies investigating this research question.

## 2 Methods

### 2.1 Study design

This pilot sibling-matched case–control study evaluated atopic outcomes in children with Down syndrome compared with their unaffected siblings. A 1:1 sibling-matched design was employed, with each child with Down syndrome paired with one unaffected sibling from the same family.

### 2.2 Study population

Children with genetically confirmed trisomy 21 born at King Khalid University Hospital (KKUH) were identified through the electronic medical records system (E-Sihi). Eligible participants were aged 4 months to 14 years and had at least one unaffected sibling in the same age range whose parents agreed to complete the questionnaire for both children. For inclusion in the analytic sample, each child with Down syndrome was required to have one completed paired questionnaire from an unaffected sibling. Questionnaires without a corresponding paired sibling or paired child with Down syndrome were excluded from the sibling-matched analysis.

### 2.3 Questionnaire and data collection

Data were collected over a two-month period using a structured Arabic-language questionnaire based on the core items of the International Study of Asthma and Allergies in Childhood (ISAAC) instrument. The selected ISAAC questionnaire items are provided in Supplementary Table S1. Parents completed the questionnaire for both their affected and unaffected children simultaneously. Clinical documentation from physician reports, when available, was reviewed to corroborate parental responses regarding respiratory and atopic profiles. Binary outcomes assessed included reported wheezing in the past 12 months, severe wheezing, defined as wheezing limiting speech or causing sleep disturbance, physician-diagnosed asthma, allergic rhinitis, and eczema.

### 2.4 Statistical analysis

All statistical analyses were performed using R version 4.5.2 (R Foundation for Statistical Computing, Vienna, Austria). Because the study employed a sibling-matched design, observations within families were not independent. Accordingly, paired statistical methods were applied for all inferential analyses.

For each binary outcome, within-pair comparisons were performed using McNemar's test, which evaluates differences within discordant sibling pairs, sibling pairs in which the outcome differs between the affected and unaffected child. Effect size was expressed as the matched odds ratio, calculated as the ratio of discordant pairs in which the outcome occurred only in the child with Down syndrome (b) to those in which it occurred only in the unaffected sibling (c).

Because this was a small pilot study in which the number of discordant pairs was expected to be limited, exact methods were used: exact McNemar *p*-values based on the exact binomial distribution, and exact 95% confidence intervals for the matched odds ratios calculated using the conditional exact method (exact 2 × 2 package in R). All tests were two-sided with a nominal significance threshold of *p* < 0.05. Given the pilot nature of the study, *p*-values were interpreted descriptively rather than as confirmatory evidence.

Baseline demographic characteristics were compared between groups using the Wilcoxon signed-rank test for age and McNemar’s exact test for sex, consistent with the matched design.

### 2.5 Ethical approval

This study received ethical approval from the Institutional Review Board at King Khalid University Hospital (No. E-24-9364). Written informed consent was obtained from parents of each participant for the use of medical information, questionnaire completion, and publication, ensuring confidentiality and anonymity.

## 3 Results

A total of 89 questionnaires were received: 54 from children with Down syndrome and 35 from unaffected siblings. For the sibling-matched analysis, 20 Down syndrome questionnaires were excluded because no paired sibling questionnaire was available, and one sibling questionnaire was excluded because no paired Down syndrome questionnaire was available. The final analytic sample therefore comprised 34 complete sibling pairs, corresponding to 68 children. The two groups were similar at baseline (Table 1). The median age was 5.0 years (IQR 3.0–7.0) in children with Down syndrome and 6.0 years (IQR 3.2–9.0) in sibling controls, with no statistically significant within-pair difference (*p* = 0.307). The median within-pair absolute age difference was 3.0 years (IQR 1.0–5.1). Male sex was reported in 17 children with Down syndrome (50.0%) and 21 sibling controls (61.8%) (*p* = 0.424). Prevalence and paired comparisons for all five outcomes are shown in (Table 2). Wheezing was the most common outcome and showed the clearest difference between groups. It was reported in 15 of 34 children with Down syndrome (44.1%) and 9 of 34 siblings (26.5%). The comparison rested on 12 discordant pairs: 9 in which only the child with Down syndrome wheezed and 3 in which only the sibling did, giving a matched odds ratio of 3.00 (95% CI 0.75–17.23). The difference favored children with Down syndrome but did not reach statistical significance (*p* = 0.146).

**Table 1:** Demographic and baseline characteristics of children with Down syndrome and sibling controls.

Characteristic	DS group (*n* = 34)	Sibling group (*n* = 34)	*p* value
Age, years, median (IQR)	5.0 (3.0–7.0)	6.0 (3.2–9.0)	0.307
Within-pair absolute age difference, years, median (IQR)	3.0 (1.0–5.1) across matched pairs		–
Male sex, n (%)	17 (50.0)	21 (61.8)	0.424

IQR, interquartile range. Age was compared within sibling pairs using the Wilcoxon signed-rank test. Sex was compared using exact McNemar testing. The within-pair absolute age difference was calculated as the absolute difference between the age of each child with Down syndrome and the matched sibling control and is reported descriptively.

**Table 2:** Paired comparison of respiratory and allergic outcomes using exact McNemar testing (*n* = 34 sibling pairs).

Outcome	DS *n* (%)	Sibling *n* (%)	Discordant pairs DS+/sib- (b)	Discordant pairs DS-/sib + (c)	Matched OR (b/c)	Exact 95% CI	Exact McNemar p
Wheeze	15 (44.1%)	9 (26.5%)	9	3	3.00	0.75-17.23	0.146
Severe wheeze	2 (5.9%)	1 (2.9%)	2	1	2.00	0.10-117.99	1.000
Physician-diagnosed asthma	8 (23.5%)	6 (17.6%)	5	3	1.67	0.32-10.73	0.727
Allergic rhinitis	3 (8.8%)	2 (5.9%)	2	1	2.00	0.10-117.99	1.000
Eczema	4 (11.8%)	4 (11.8%)	2	2	1.00	0.07-13.80	1.000

DS, Down syndrome; OR, odds ratio; CI, confidence interval. Paired comparisons were performed using exact McNemar testing. The matched odds ratio was calculated from discordant pairs as b/c, where b represents pairs in which only the child with Down syndrome had the outcome and c represents pairs in which only the sibling control had the outcome. Exact 95% confidence intervals were calculated using conditional exact methods.

Physician-diagnosed asthma followed the same direction. It was reported in 8 children with Down syndrome (23.5%) and 6 siblings (17.6%), with 5 discordant pairs favoring the child with Down syndrome and 3 favoring the sibling (matched odds ratio 1.67, 95% CI 0.32–10.73; *p* = 0.727).

**Figure 1:**
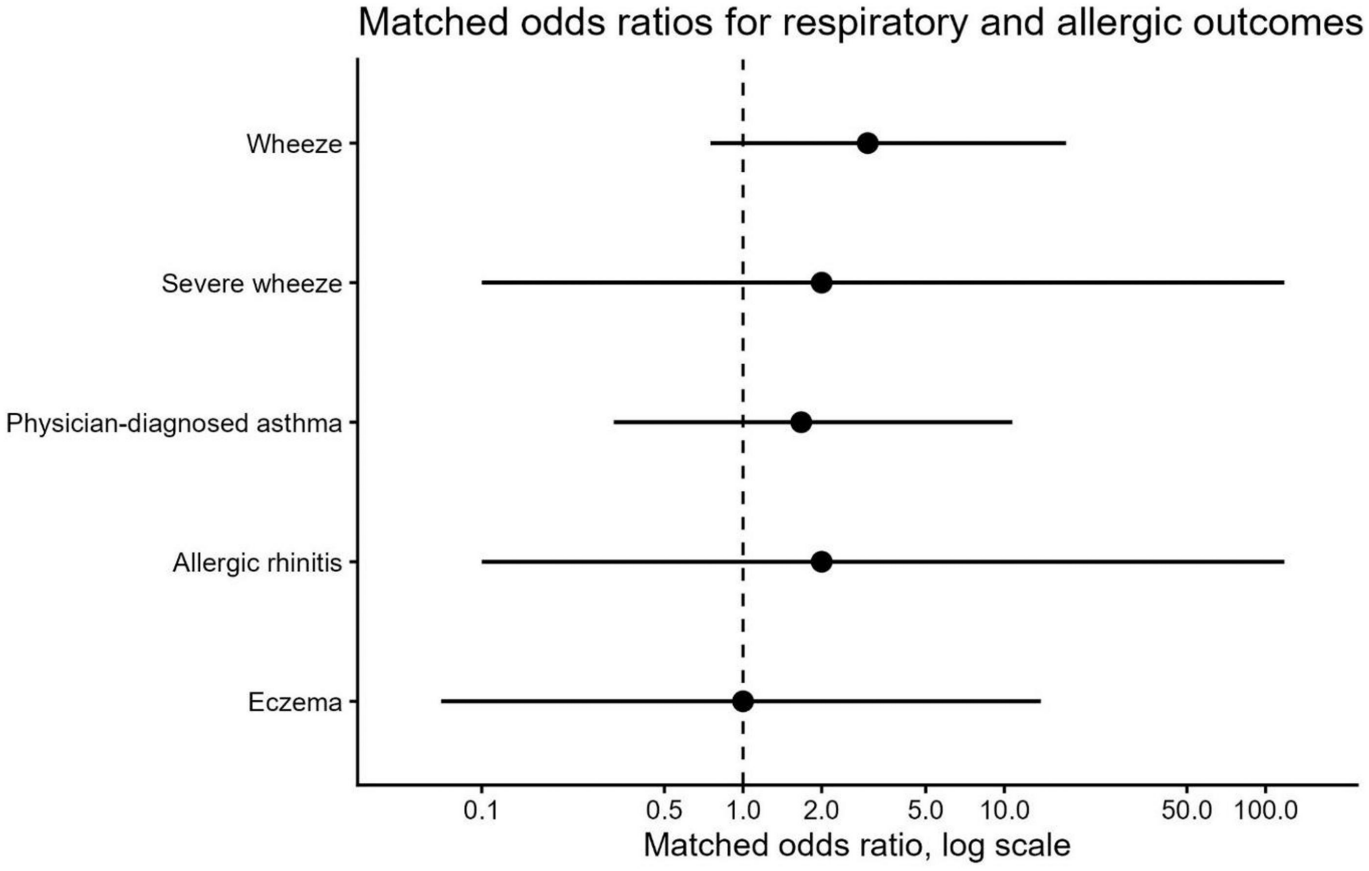
Matched odds ratios for respiratory and allergic outcomes in children with Down syndrome compared with sibling controls.

The remaining outcomes were uncommon and showed little or no difference. Severe wheezing occurred in 2 children with Down syndrome (5.9%) and 1 sibling (2.9%) (matched odds ratio 2.00, 95% CI 0.10–118; *p* = 1.00). Allergic rhinitis was reported in 3 children with Down syndrome (8.8%) and 2 siblings (5.9%) (matched odds ratio 2.00, 95% CI 0.10–118; *p* = 1.00). Eczema was equally common in both groups, affecting 4 children (11.8%) in each, with evenly distributed discordant pairs (matched odds ratio 1.00, 95% CI 0.07–13.80; *p* = 1.00). Across all five outcomes, the confidence intervals were wide and crossed the null value of 1, reflecting the small number of discordant pairs available for each comparison (Figure 1).

## 4 Discussion

In this pilot sibling-matched study, children with Down syndrome had numerically higher frequencies of wheezing and physician-diagnosed asthma than their unaffected siblings; however, these differences did not reach statistical significance, and the confidence intervals were wide. The main contribution of this study is methodological: it demonstrates the feasibility of a sibling-matched design analyzed with appropriate paired methods and provides preliminary effect-size estimates to inform future adequately powered studies. Anatomical abnormalities, immunological alterations, and increased susceptibility to respiratory infections may plausibly predispose children with Down syndrome to wheeze (4, 5).

A local study assessing respiratory symptoms among 115 children with trisomy 21 reported wheezing in 47.8% of participants regardless of the underlying aetiology (9), a rate comparable to the 44.1% prevalence of wheeze we observed among children with Down syndrome.

In a related sibling-comparison study using a different statistical approach, Weijerman et al. assessed wheeze ever and wheeze during the preceding 12 months among children with Down syndrome, their siblings, and population controls. They reported a relative risk of current wheeze of 2.8 compared with siblings and 2.75 compared with population controls. In contrast, our study focused on the sibling-matched comparison and used exact McNemar testing to account for within-family pairing. We observed a matched odds ratio of 3.00 for wheezing, derived from the discordant pairs: 9 pairs in whch only the child with Down syndrome wheezed versus 3 pairs in which only the sibling did. However, the paired comparison did not reach statistical significance, likely reflecting the limited sample size and small number of discordant pairs (4).

In one report, children with DS were less likely to have received a doctor's diagnosis of asthma (3.1%) than siblings (4.2%) or controls (6.7%, *p* = 0.04) (4). In the population-based National Health Interview Survey analysis by Schieve et al., the sample included 146 children with Down syndrome and 95,454 children without Down syndrome or intellectual disability. Children with Down syndrome had increased odds of asthma that approached statistical significance compared with children without intellectual disability, although medical conditions were reported by parents or other knowledgeable caregivers rather than confirmed through standardized clinical assessment (10). Chronic rhinitis occurred more frequently in children with DS (40%) than in their siblings (17.3%); eczema did not (14.6 vs 19.2%) (4). Given our small sample size, eczema and allergic rhinitis were comparable in our subjects despite the general type-2 (Th2) inflammation biology that likely applies in DS, superimposed on DS-specific immune abnormalities compared with typically developing peers (5, 11, 12).

Another cohort concurred with known facts of wheeze and its recurrence being more commonly prevalent in children with DS compared to healthy controls including siblings regardless of the respiratory syncytial Virus (RSV) status (13). The frequency of respiratory symptoms and length of stay have also been described as higher in DS children compared to the general population (5). Although this variable was not directly assessed in our study, these findings collectively support the observation that children with Down syndrome experience more frequent and prolonged respiratory symptoms.

Within families, wheezing was numerically more frequent among children with Down syndrome than among their unaffected siblings, although this difference did not reach statistical significance and the confidence intervals were wide. Because the matched comparison depends on the small number of discordant pairs, these estimates are imprecise and should be regarded as hypothesis-generating. Mechanisms such as airway anomalies and immune dysregulation in Down syndrome may contribute to susceptibility and merit further investigation.

This study has several limitations. First, the small sample size of 34 sibling pairs limited statistical power, and none of the paired comparisons reached significance despite numerically higher frequencies of wheezing and asthma among children with Down syndrome. The confidence intervals were wide and crossed the null value of 1, supporting descriptive rather than confirmatory interpretation. Second, we did not prespecify a standardized sibling-selection criterion for families with multiple unaffected siblings. Future studies may benefit from predefined matching strategies, such as selecting the closest-age or immediately older sibling, to improve consistency across paired analyses. Third, the questionnaire was built around selected core items of the ISAAC instrument, which was developed and validated primarily for school-age children and was not formally validated across the full age range included in this study; several severity items, such as wheeze limiting speech, are difficult for parents to assess in the youngest participants. Future studies should use age-appropriate instruments or item subsets across the pediatric age range.

Despite these limitations, this study illustrates the value of applying a paired analytical framework using exact McNemar testing and matched odds ratios within a sibling-matched design evaluating atopic outcomes in Down syndrome. Previous sibling-matched studies used unpaired tests that ignored within-family correlation. This methodological contribution provides a template and preliminary effect-size estimates to inform the design and sample-size planning of future adequately powered studies.

## 5 Conclusion

In this pilot sibling-matched study, wheezing and physician-diagnosed asthma were numerically more frequent among children with Down syndrome compared with unaffected siblings. However, none of the paired comparisons reached statistical significance, likely reflecting the limited number of discordant pairs and the exploratory nature of the study. Importantly, these findings highlight that statistical comparisons in sibling-matched designs are driven primarily by discordant pairs rather than overall prevalence estimates, underscoring the importance of paired analytical approaches as a methodological framework for future adequately powered studies investigating this research question.

## Data Availability

The datasets presented in this article are not readily available because none requests to access the datasets should be directed to nsalharbi@ksu.edu.sa.
