# Supplementary material for: Atopic outcomes in children with Down syndrome: a pilot sibling-matched case–control study
Source: Front Pediatr. 2026 Jul 24;14:1883821. doi: 10.3389/fped.2026.1883821 (PMC13447469; doi:10.3389/fped.2026.1883821)
Supplement: Supplementary file 1 [file Table1.docx]

Supplementary Table S1. Selected ISAAC questionnaire items used to assess respiratory and atopic outcomes and their clinical interpretation

| Question | Answer | Interpretation |
| --- | --- | --- |
| Asthma | | |
| Wheeze in past 12 months? | Yes  No | Suggests current asthma symptoms.  Less likely active asthma |
| Ever had asthma? | Yes  No | Lifetime prevalence of asthma.  No reported history |
| Number of wheezing attacks in past 12 months? | 1-3  4-12  > 12 | Mild asthma.  Moderate asthma.  Severe asthma, consider urgent intervention. |
| Wheeze causing sleep disturbance? | Yes  No | Indicated more severe asthma.  Mild, or well-controlled asthma. |
| Wheeze limiting speech? | Yes  No | Severe asthma, an emergency risk.  Mild-to-moderate asthma. |
| Dry night cough apart from cold? | Yes  No | Supports asthma diagnosis.  Less likely asthma. |
| Eczema | | |
| Ever had itchy rash lasting 6 months? | Yes  No | Suggests lifetime eczema.  Less likely eczema. |
| Rash in past 12 months? | Yes  No | Current eczema.  Not actively current. |
| Rash in typical areas (Elbows, knees, wrists, ankles)? | Yes  No | Classic atopic dermatitis.  Could indicate other skin conditions. |
| Allergic rhinitis | | |
| Ever had sneezing/runny/blocked nose without cold? | Yes  No | Suggests allergic rhinitis.  Less likely. |
| Symptoms in past 12 months? | Yes  No | Current allergic rhinitis.  Not active. |
| Accompanied by itchy-watery eyes? | Yes  No | Confirms allergic rhino-conjunctivitis.  Isolated rhinitis. |

**Note:** the questionnaire was a structured Arabic-language tool built around the core items of the International Study of Asthma and Allergies in Childhood (ISAAC) instrument, rather than a formally validated ISAAC version. ISAAC was originally developed and validated for school-age children and is not specifically validated across the full pediatric age range; items were therefore used to support descriptive interpretation of respiratory and atopic symptoms in this pilot.
